# ATR-FTIR spectroscopy of plasma supported by multivariate analysis discriminates multiple sclerosis disease

**DOI:** 10.1038/s41598-023-29617-6

**Published:** 2023-02-13

**Authors:** Maria Caterina Crocco, María Fernanda Heredia Moyano, Ferdinanda Annesi, Rosalinda Bruno, Domenico Pirritano, Francesco Del Giudice, Alfredo Petrone, Francesca Condino, Rita Guzzi

**Affiliations:** 1 Molecular Biophysics Laboratory, Department of Physics, University of Calabria, 87036 Rende, Italy; 2 STAR Research Infrastructure, University of Calabria, 87036 Rende, CS Italy; 3 CNR-Nanotec Rende, Via P. Bucci, 87036 Rende, Italy; 4 Department of Pharmacy, Health and Nutritional Sciences, University of Calabria, 87036 Rende, CS Italy; 5 Neurological and Stroke Unit, Multiple Sclerosis Clinic, Annunziata Hospital, 87100 Cosenza, Italy; 6 Department of Economics, Statistics and Finance “Giovanni Anania”, University of Calabria, Arcavacata di Rende, CS Italy; 7 Present Address: SOC Neurologia-Azienda Ospedaliera Pugliese-Ciaccio, 88100 Catanzaro, Italy; 8 Present Address: SOC Neurologia-Ospedale Jazzolino, Azienda Ospedaliera Provinciale, 89900 Vibo Valentia, Italy

**Keywords:** Biophysics, Biomarkers, Diseases

## Abstract

Multiple sclerosis (MS) is one of the most common neurodegenerative diseases showing various symptoms both of physical and cognitive type. In this work, we used attenuated total reflection Fourier transformed infrared (ATR-FTIR) spectroscopy to analyze plasma samples for discriminating MS patients from healthy control individuals, and identifying potential spectral biomarkers helping the diagnosis through a quick non-invasive blood test. The cohort of the study consists of 85 subjects, including 45 MS patients and 40 healthy controls. The differences in the spectral features both in the fingerprint region (1800–900 cm^−1^) and in the high region (3050–2800 cm^−1^) of the infrared spectra were highlighted also with the support of different chemometric methods, to capture the most significant wavenumbers for the differentiation. The results show an increase in the lipid/protein ratio in MS patients, indicating changes in the level (metabolism) of these molecular components in the plasma. Moreover, the multivariate tools provided a promising rate of success in the diagnosis, with 78% sensitivity and 83% specificity obtained through the random forest model in the fingerprint region. The MS diagnostic tools based on biomarkers identification on blood (and blood component, like plasma or serum) are very challenging and the specificity and sensitivity values obtained in this work are very encouraging. Overall, the results obtained suggest that ATR-FTIR spectroscopy on plasma samples, requiring minimal or no manipulation, coupled with statistical multivariate approaches, is a promising analytical tool to support MS diagnosis through the identification of spectral biomarkers.

## Introduction

Multiple sclerosis (MS) is a chronic inflammatory neurodegenerative disease of the central nervous system (CNS) causing demyelination of neurons and axonal loss. It affects mostly young adult, ultimately leading to long term disability^1^. MS prevalence estimated worldwide is about 2.8 million people in 2020, prevailing in more developed countries, high-income classes, and with women showing a greater propensity (female to male ratio is 3 to 1) for the disease^2^.

On a medical basis, a wide variety of neurological signs and symptoms may occur alone or in combination with a recurring trend including sensory disturbances, motor weakness, visual complaints, incoordination, fatigue, spasticity, sleep disorder, sexual dysfunction^3^. MS diagnosis is not easy, and its misdiagnosis, quoted in about 10%, remains a persistent problem with potential consequences for both patients and health systems^4,5^. There is no single pathognomonic clinical feature or diagnostic test of MS based on the integration of clinical, imaging and laboratory evidences, according to the McDonald criteria revised in 2017, and stress the need for no better explanation to account for the variety of symptoms observed^6^. These criteria include typical MRI pattern showing CNS lesions disseminated in time and space, cerebrospinal fluid (CSF) analysis demonstrating increase in immunoglobulin (IgG) concentrations and the presence of oligoclonal bands, delayed latencies of visual evoked potentials. In contrast, blood tests are commonly used rather to rule out other diseases.

The clinical course of MS is highly variable and largely unpredictable. Clinical isolated syndrome and relapsing–remitting (RR) form represent the very early stage of the disease. Recurring relapses generally coincide with inflammation and demyelination of CNS area. As a self-sustaining inflammation occurs and remyelination is inhibited, neurodegeneration progresses and patient disability accumulates (secondary progressive, SP, phase of the disease). In about 15% of patients, the course of the disease may be characterised by a gradual progression from the onset (primary progressive MS). Considerable disease activity may also occur within the CNS in the absence of clinical symptoms^4,6^. The expanded disability status scale (EDSS), is the most common clinical outcome measure that integrates neurological findings in eight functional systems, and mainly relies on the assessment of the patient walking ability. The EDSS values ranges from 0 to 10, corresponding to a normal neurological status or to death of the patient due to MS, respectively. EDSS score greater than 7 indicates a very compromised clinical status.

The complexity of the MS disease pushes in searching new strategies to combine current diagnostic methods with advanced complementary approaches able to identify and quantify new significant parameters, especially at the beginning of the disease, when the pathological signs may be mild and/or not very specific.

An emerging field of interest in clinical investigation is the search of novel biomarkers/metabolites to be used as supporting tools in diagnosis and monitoring the disease progression. For MS, CSF represents the ideal source of biomarkers^7–9^, given its proximity to CNS, but lumbar puncture is an unpleasant procedure for the patient and is therefore not an optimal tool. In contrast, blood samples can be easily collected and may reflect the status of both peripheral immune system and, indirectly, of CNS functioning mechanisms^7,10,11^. In fact, blood perfuses all body organs and thus contains a large range of biomolecules from surrounding tissues and cells, carrying information on intra- and extracellular events^12^. Moreover, 0.5 L of CSF is adsorbed into the blood every day, suggesting that plasma may be a source of disease biomarkers originating from CSF, too^13^. The ability to qualitatively and quantitatively characterize biofluids is extremely valuable in a clinical contest. To detect and quantify biological materials such as proteins, carbohydrates, nucleic acids, lipids, metabolites, as well as searching for disease-specific biomarkers, high sensitivity biophysical/analytical techniques are available. These methods include differential scanning calorimetry^14–16^, mass spectrometry^17^, nuclear magnetic resonance (NMR)^18,19^ and infrared (IR) spectroscopy^20^. In particular, vibrational spectroscopy, in the mid-IR range, is largely expanding in the biomedical area to investigate complex biological fluids, such as blood and blood derivatives (plasma and serum), CSF, saliva, as well as cells and tissues^20–24^. A notable advantage of the technique is the possibility to investigate simultaneously the presence of different biomolecules contained in the samples. IR spectroscopy, may be applied in different modality, including attenuated total reflection (ATR) and transmission, allowing one for a rapid and label-free investigation of samples with minimal or no manipulation. Interestingly, comparison of spectral profiles of biological samples from healthy or diseased individuals has revealed, often supported by multivariate statistical analysis, distinctive and quantifiable changes contributing to validate Fourier transform IR (FTIR) spectroscopy as a complementary non-invasive tool for the diagnosis and discrimination of several malignancies. These pathologies include neurodegenerative diseases^25^, different types of cancer (melanoma, breast, brain)^12^, COVID-19^26,27^, systemic amyloidosis^28^.

In the present work we use ATR-FTIR spectroscopy to analyze plasma samples for discriminating MS patients from healthy individuals, in combination with multivariate statistical methods. The analysis of the results shows significant differences of specific spectral regions related to functional groups of proteins and lipids in the two groups of individuals. The potential spectral markers were identified by multivariate statistical analysis.

## Materials and methods

### Subjects

MS patients were recruited from April 2017 to July 2018 in the MS Center of the Annunziata Hospital in Cosenza, Italy, and examined by neurologist to evaluate the degree of progress of the disease. Inclusion criteria were: age in the range 18–70 years and a confirmed MS diagnosis according to the revised McDonald criteria^6^. Exclusion criteria were: concomitant autoimmune disorders other than MS; pregnancy; and a high degree of cognitive decline preventing the expression of an informed consent. The collected clinical and personal data of the patients included: gender, age, disease duration, disease severity expressed in terms of EDSS, and current pharmacological treatment.

The MS group consisted of 45 patients, including 31 females and 14 males, with an average age of 42.7 years (range 22–69 years). As regards the clinical form of the pathology, for most of the patients the diagnosis was RRMS and few of them (7 patients) were in the SPMS phase. The time from the onset of the disease was very variable, ranging from 1 to 47 year and in particular: 37.7% ≤ 5 years, 22.2% in the range 6–10 years, 20% 11–15 years, 11.1% 16–20 years, 6.6% 24–35 years, and one patient with a diagnosis dating back 47 years. All the patients, with the exception of seven of them, were treated with immuno-modulatory or suppressive therapy. The EDSS values in the MS cohort ranged from 0.5 to 7.0 (values 0.5–3.0 are considered mild disability, 3.5–7.0 are considered as moderate/severe disability). Most of the patients (32 individuals) had EDSS score in the 0.5–3.0 range, whereas 13 patients belonged to the 3.5–7.0 range.

Control population consisted of 40 healthy control (HC) individuals, including 21 females and 19 males. They were recruited in part (21 subjects) in the same Annunziata Hospital in Cosenza, and in part (19 subjects) among blood donors at the Centro Sanitario of the University of Calabria, Italy. The average age was 37.3 years (range 24–60 years, one outlier of 71 years), and all of them showed no evidence of inflammatory and neurological diseases. Table 1 summarize the demographic and clinical data of the individuals participating to the study. The chi-square and the t-test p-values obtained from the comparison of sex and age, respectively, between MS and HC are also given.

**Table 1:** Demographic data and clinical variable of the MS patients.

	MS patients	HC	p-value
Gender			0.1853
Female, F	31	21	
Male, M	14	19	
Total	45	40	
Mean age (years)	42.7 ± 11.3	37.3 ± 10.9	0.029
EDSS	2.44 ± 1.71		
0.5–3.0 (32 subjects, mild)	1.48 ± 0.64		
Gender 22 F + 10 M			
3.5–7.0 (13 subjects, moderate/severe)	4.81 ± 1.07		
Gender 9 F + 4 M			
Disease onset			
≤ 10 years	5.26 ± 2.55		
Gender 17 F + 10 M			
> 10 years	18.39 ± 9.47		
Gender 14 F + 4 M			

Values are quoted as average ± standard deviation.

The components of both groups had the same ethnic origin (Calabria, southern Italy). Each subject recruited was fully informed about the purpose of the study and a written consent was acquired. The study was approved by the Ethics Committee of the north area of the Calabria region (protocol n. 50 of February 14, 2017). All methods were performed in accordance with the relevant guidelines and regulations.

### Blood sample processing

Samples (3 mL) of peripheral venous blood were collected in EDTA tubes, and plasma was separated by centrifugation for 15 min at 1500 rpm. Processed plasma was dispensed in 0.15 mL aliquot and stored at − 20 °C until use.

### ATR-FTIR measurements

After thawing, plasma samples were degassed at 25 °C before measurements. Spectra were acquired with a Tensor II spectrometer from Bruker (Bruker Optics, Germany) equipped with a silicon BioATR II cell optimized for liquid samples and an MCT detector. The cell was thermally controlled by a PilotOne thermostat. 20 µL of plasma were deposited in the sample cell and left to equilibrate for 3 min before measurements performed at constant temperature, 25 °C. A background spectrum was recorded before every acquisition using Dulbecco Phosphate Buffer Saline (DPBS) solution, 10 mM at pH 7.4 (from Sigma Aldrich, St. Louis, Missouri, USA) and was automatically subtracted from the sample spectrum. The spectra, recorded in the 4000–900 cm^−1^ range with a spectral resolution of 4 cm^−1^, were averaged over 120 scans. For each sample up to five replicate spectra were collected to asses reproducibility.

### Multivariate and univariate statistical analysis

Before applying the statistical analysis, the absorption spectra were pre-processed using the Bruker Opus software version 7.0 as follows: cut of the fingerprint region (1800–900 cm^−1^) and high region (3050–2800 cm^−1^), rubber band baseline correction, vector normalization. The absorption spectra recorded for each sample were averaged in order to compare individuals rather than spectra. Then the 40 HC and 45 MS ATR-FTIR spectra obtained were further averaged to obtain the mean and the standard deviation of each class. Averaged spectra minimize the influence of individual differences and thus are more representative.

Second-order derivatives of the mean spectra were calculated using the Savitsky-Golay algorithm with 13 smoothing points. For the second derivative spectra, vector normalization was the final step of the correction^20,25^.

Statistical spectral differences of specific bands in the absorbance of HC and MS groups were assessed using two tailed parametric t-test or one-way ANOVA, followed by unpaired t-test corrected according to Bonferroni for multiple comparisons by using GraphPad Prism, version 9.3.1 (GraphPad Software, www.graphpad.com). A p-value of 0.05 or less was considered significant in all statistical tests. The degree of significance for the comparison of the data with the control group were indicated as *p < 0.05; **p < 0.01; ***p < 0.001; ****p < 0.0001.

Multivariate data analysis was performed by using R Statistical Software, version 4.1.0 (R Core Team 2021)^29^ on both absorption and second derivative spectra. In particular, the *caret* package was used to implement the multivariate models. The second derivative was then preferred in the subsequent analysis, since it allows one for better resolution of overlapping peaks and accounts for spectral differences in more detail. In this study, three different multivariate data analysis methods have been considered: principal component linear discriminant analysis (PCA-LDA), partial least-squares discriminant analysis (PLS-DA) and random forest (RF).

PCA is an unsupervised technique used for dimensionality reduction. Each data point is projected onto subspace identified only by the first few principal components, with the aim to obtain lower-dimensional data while preserving as much of the data variation as possible. In PCA-LDA model, the variables corresponding to the wavenumbers in the considered spectral regions were reduced into few PCs that represented > 95% of the variance. The scores of data points are then used as input data for linear discriminant analysis to maximize the distance between the MS and HC groups.

Partial least squares discriminant analysis (PLS-DA) was proposed by Pérez-Enciso and Tenenhaus^30^ as a compromise between the conventional discriminant analysis and a discriminant analysis on the most significant principal components of the predictor variables. It is a variant of the partial least squares regression, able to take into account the binary nature of the response variables. Moreover, this technique is especially suited to deal with a large number of predictors and with multicollinearity, as in the present case.

Random forest is a supervised algorithm widely used in classification and regression problems. First proposed by Ho^31^ and extended by Breiman^32^, it builds a multitude of decision trees on different randomly chosen samples and, for classification tasks, takes the class selected by most trees as final prediction. A detailed description of the algorithm implemented in *caret* package of *R* and used in this work can be found in Liaw and Wiener^33^.

For all the three statistical techniques, we performed a double step of validation. In the first step, was considered an internal validation based on 10-time repeated five-fold cross validation data to select the value of tuning parameter, when present, and to assess the predictive discrimination of the model. To this end, only 75% of the original data was used as training set. Specifically, in the PLS-DA and RF approaches, we considered the number of latent variables and the number of variables randomly collected to be sampled at each split time, respectively, as tuning parameter. Once this value was selected, the final model was refit on the whole training set. The remaining 25% of data was used in the second step, as test set for an external validation procedure and to compare the final models obtained from the previous step.

The fraction of the correct predictions, namely the accuracy, was used to evaluate each classification model and to select the best one. The performance of the final classification model was evaluated on the test set considering different measures based on the confusion matrix, such as the sensitivity and specificity of the method, defined as:

$$ {\text{Sensitivity }} = \frac{TP}{{TP + FN}} $$

$$ {\text{Specificity }} = \frac{TN}{{TN + FP}} $$

where TP, TN, FP and FN refer to true positives, true negatives, false positives and false negatives. Moreover, the Receiver Operating Characteristic (ROC) curve, a plot of sensitivity as a function of specificity for different cut-off points of test class probabilities, was determined. The area under this curve (AUC) was taken as a measure of how well the final model can distinguish between MS and HC.

To characterize the general effect of predictors on the model and to determine the different IR wavenumbers importance, a measure of variable importance is obtained, according to the specific method. In particular, for the PLS-DA model the variable importance is obtained considering a weighted sum of the absolute regression coefficients, where the weights depend on the reduction of the sums of squares across the number of PLS components. The variable importance in the RF model is obtained by following the permutation principle of the ‘mean decrease in accuracy’ importance. For each tree, the prediction accuracy on the out-of-bag portion of the data is recorded. Then, the same procedure was applied after permuting each predictor variable. The difference between the two accuracies was then averaged over all trees and normalized by the standard error. Finally, for PCA-LDA model, the variable importance was obtained by considering the approach based on the ROC curve, and the AUC was used to quantify the predictor relevance. Indeed, if a predictor is able to perfectly separate HC from MS, there will be a cut-off for which both sensitivity and specificity will be equal to 1, as well as the AUC. Therefore, for each predictor a series of cut-offs were applied, and the sensitivity and specificity were computed. Then, the trapezoidal rule was used to compute the AUC, taken as a measure of variable importance.

The final model obtained from each method was also used to evaluate the prediction for patients having different disability degree, distinguishing from mild (EDSS ≤ 3) and moderate/severe disability (EDSS > 3) as well as short (≤ 10 years) or long disease duration (> 10 years).

## Results and discussion

### Spectral analysis of MS and HC plasma samples

In this study we performed ATR-FTIR investigation of plasma samples from 45 patients affected by multiple sclerosis with different degree of disability, 32 subjects have an EDSS value ≤ 3 (mild disability) and 13 are characterized by EDSS values between 3.5 and 7.0 (moderate/severe disability). The infrared spectra are compared with a control population not affected by inflammatory or neurological diseases. The main demographical information about the two groups of subjects are listed in Table 1.

**Figure 1:**
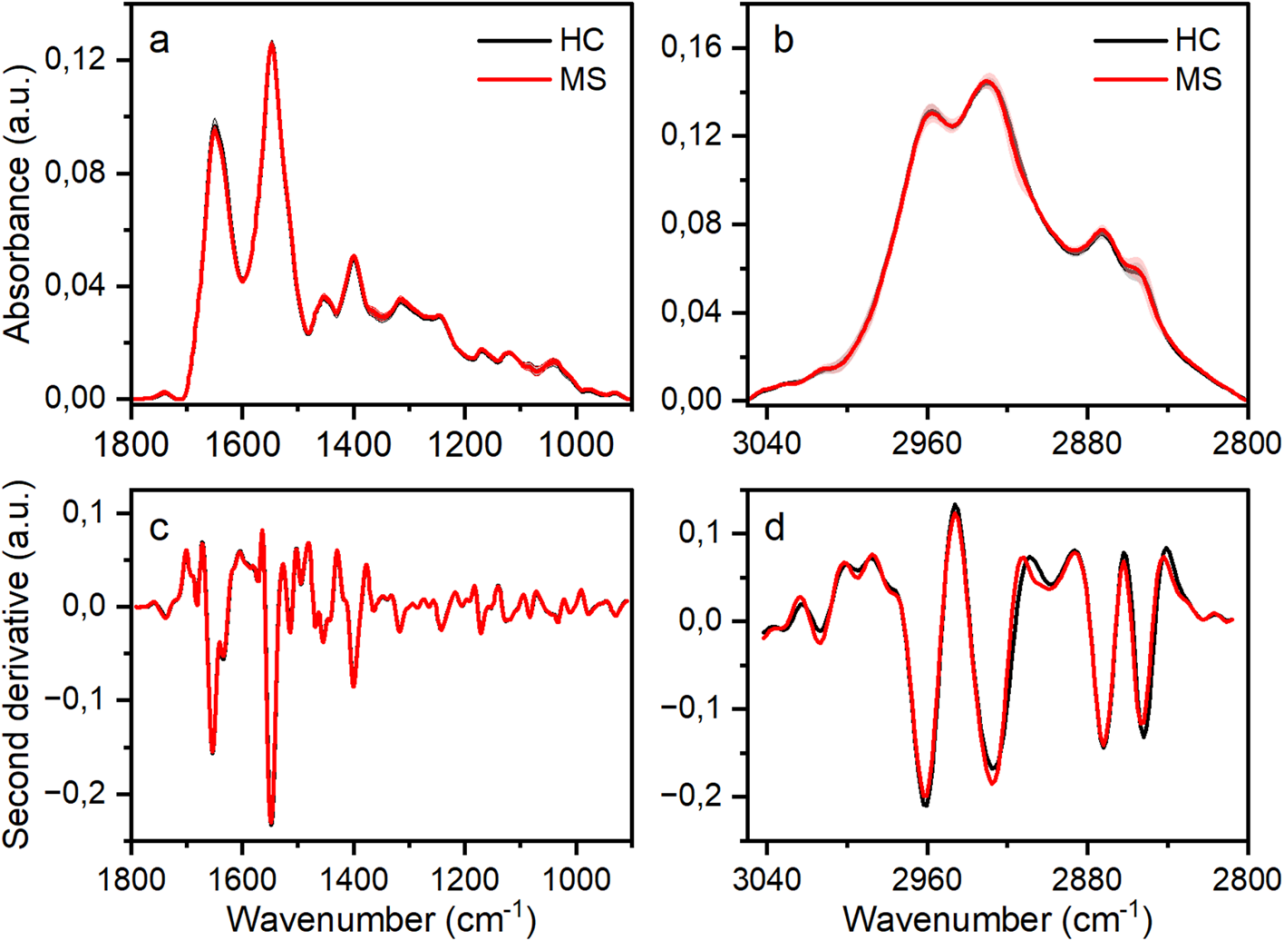
ATR-FTIR average spectra, recorded at 25 °C, of plasma samples from 40 HC (black line) and 45 MS (red line) subjects. Mean absorbance spectra in the (**a**) fingerprint region, 1800–900 cm^−1^ and (**b**) high region, 3050–2800 cm^−1^. In the (**c**, **d**) panels are shown the second derivative vector normalized spectra.

The infrared spectra of plasma samples were acquired at 25 °C in the 4000–900 cm^−1^ spectral range for the healthy control individuals and MS patients. Figure 1 reports the mean absorbance spectra and the corresponding second derivative ones obtained for the two classes of samples in the fingerprint region, 1800–900 cm^−1^ (panels a, c), and in the high region, 3050–2800 cm^−1^ (panels b, d). Second derivative of the absorbance spectra are normally used to separate overlapping adjacent bands.

The first region mainly corresponds to the vibration of the functional groups of proteins, with the two peaks of higher intensity assigned to the amide I (1650 cm^−1^, C=O stretching vibration) and amide II band (1547 cm^−1^, N–H bending and C–N stretching). The proteomic profile of blood plasma is very complex and contains contributions of more than 20,000 different proteins^34^ covering a large range of concentrations (pg-mg/mL), that correlates with the spectroscopic response/sensitivity. The proteins with a higher abundance in plasma are human serum albumin (HSA ~ 60% by weight), immunoglobulin (IgG ~ 14%), transferrin (6%), and fibrinogen (4%). HSA has a prevalent α-helix secondary structure and account for the peak at 1650 cm^−1^, whereas IgG is a β-sheet protein absorbing at about 1633 cm^−1^ (Fig. 1, panel c). In the fingerprint region, lipids are the main contributors to the bands centered at 1740 and 1453 cm^−1^ (see Table S1 in the supplementary information for bands assignment).

The high region of the ATR-FTIR spectrum (Fig. 1, panel b) is mainly assigned to the asymmetric (2960 and 2927 cm^−1^, respectively) and symmetric (2872 and 2852 cm^−1^, respectively) stretching vibrations of CH_3_ and CH_2_ groups in lipids and proteins. Also contributing in this region are the N–H (${NH}_{3}^{+}$) stretching vibrations^35–37^.

The lipidomic profile in plasma include both free lipids (cholesterol, triglycerides, phospholipids, fatty acids) and protein-bound lipids. Lecithin, sphingomyelin and lysolecithin are the three major phospholipids in normal human plasma, accounting for 95% on the basis of their weight fraction.

In both fingerprint and high regions, the spectral features of the HC and MS average spectra are very similar, and from visual inspection alone it is difficult to detect specific differences that may be related to the health status. Similar findings were recently found in preliminary FTIR studies performed on blood fractions of MS patients^38,39^. This result is expected, since plasma and serum components are common in all individuals, regardless whether they are diseased or healthy. However, slightly changes in the intensity of specific peaks are observed (see the enlargements in Figs. S1, S2) across the average spectra that can be related to variations in the concentration of the functional groups belonging to the corresponding molecular component through the Lambert–Beer law^35^. In particular, the 1750–1725 cm^−1^ region assigned to the ester C=O stretching band of lipids is more intense in the MS group (p < 0.0001) whereas the amide I peak has a reduced intensity (p < 0.0001). This result indicates an alteration in the lipid and protein concentration/metabolism.

Interestingly, the C=O band has a reduced intensity in the CSF^22^ of MS patients as well as in tissue samples of grey matter and plaques from the central nervous system^40^. Such a reduction was explained in terms of a reduction of the lipid content as a consequence of the demyelination process. Myelin sheath has a high proportion of lipids (70–85% of dry weight) and is rich of cholesterol, phospholipids and glycolipid in a percentage ratio of 40:40:20^41^. The degraded/free lipids may pour into the circulatory system due to the close proximity. A recent study reports an increase of lipids within lipoproteins in MS patients compared to healthy controls^42^. Moreover, the increase in the intensity of the C=O band with respect to the intensity of the CH_2_ absorption peak at 1468 cm^−1^ (I_1739_/I_1468_, Fig. S3) could be also associated to the presence of oxidized lipids produced by free radicals, which are hypothesized to play a role in the pathogenesis of multiple sclerosis^43^. An altered lipidomic profiles is an hallmark of MS, and several studies aimed to identify possible lipid biomarkers for diagnosis and monitoring treatments by using plasma^42^, serum^44^ and CSF^45^. It is worthwhile to remember that lipids, in addition to being fundamental components of biological membranes, also play key roles in the signaling and regulation of the immune system^46^.

The albumin and IgG quotient related to their concentration in serum and CSF is a measure of the blood-CSF dysfunction, and has been used and an indicator of the blood–brain barrier (BBB) integrity. In fact, BBB leakage is a common pathological feature in MS and can results in the extravasation of the plasma components through the damaged vessels and entry into the CNS^47,48^. The occurrence of this event could explain the observed reduction in intensity of the amide I band in MS patients.

In the high region of the ATR-FTIR spectra (Fig. 1, panel d; Fig. S2) slight changes in the peak’s intensity are also evident. In particular, the band at ~ 3013 cm^−1^, assigned to the olefinic C=CH stretching vibration, is typical of unsaturated lipid and increases in MS patients (p < 0.001). The same band has a reduced intensity in the CSF^22^. Also, the intensity of the CH_2_ asymmetric stretching at ~ 2927 cm^−1^ increases in MS patients compared to healthy control individuals (p < 0.001).

**Figure 2:**
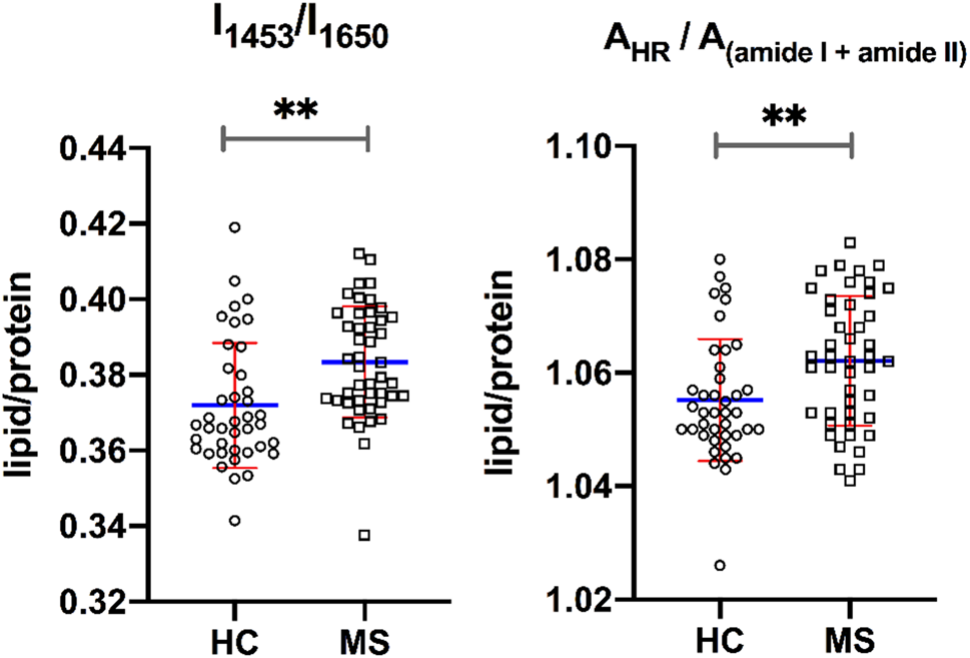
Lipid/protein ratio for HC and MS groups, obtained by taking the ratio of the intensity of the peaks at 1453 cm^−1^ to the amide I at 1650 cm^−1^ or the ratio of the areas under the high region (3050–2800 cm^−1^) and the sum under the amide I and amide II bands. The blue lines represent the mean values and the red lines the standard deviation. The degree of significance of the comparison between HC and MS is indicated as **p < 0.01.

To explore in more details the possible disease-induced differences of significant spectral bands, we have determined (1) the intensity ratio of specific functional groups, and (2) the areas ratio of molecular components. The lipid and protein components may be considered as main source of variation in the FTIR spectral features of plasma, and our analysis may capture lipid and protein dysregulation occurring in MS patients^42^. In particular, the lipid/protein ratio was determined in two different ways: by taking the ratio of the intensity of the peaks at 1453 cm^−1^ and amide I (1650 cm^−1^) or, alternatively, as the ratio of the areas under the 3050–2800 cm^−1^ region (A_HR_) and the sum of the area under the amide I and amide II bands (Fig. 2). Both determinations show an increase in the lipid/protein ratio in the MS patients compared to the control group. This finding is consistent with several studies reporting an increase in the lipids level in the serum and plasma of patients with MS^42,49–51^. A recent FTIR study performed on both plasma and serum of a limited number of MS patients reports an increase of the lipid component and a decrease of the protein component in the serum biofluid compared to the healthy control group which agrees with ours findings^39^.

We also performed statistical analysis on other possible diagnostic indicators such as bandwidth or shift in the bands position originating from lipids and proteins, but we found no statistically significant differences between healthy individuals and MS patients, denoting no measurable alteration in the conformation of these molecular components.

Patients were further separated in terms of disease severity and duration (Table 1). For disease severity we consider patients with EDSS in the mild (0.5–3.0) or in the moderate/severe (3.5–7.0) range. The comparison of the two MS subgroups with the control group provided a p-value of 0.004. For disease duration we considered short-term (≤ 10 years) and long-term (˃ 10 year) patients and in this case, we obtained a p-value of 0.005. The results on the lipid/protein ratio maintain statistical significance for MS patients having a mild disability score compared to HC and for the disease duration subgroups (Fig. S4). The biomolecular variations are revealed with higher statistical significance for patients characterized by mild EDSS score and short onset time of the disease. A possible explanation for this finding could be that at earlier stage of MS the metabolic disorder determined by inflammation in the CNS are considerable, and reflect to a larger extent in the plasma components. In contrast, at a later stage of the disease the organism tends to reach an adaptation and compensation status of the metabolic changes induced by MS, which reflect a lower degree of alteration of blood plasma^50–52^.

Sex is also an important variable in MS. However, male vs female subgroups separation does not provide statistical differences for the lipid/protein ratios (p-value ranges between 0.527 and 0.923).

### Multivariate analysis of the ATR-FTIR spectra

To uncover further subtle differences among the spectra registered for the two classes of subjects, multivariate approaches were applied. The spectra contain molecular information on the plasma components and, to capture the variables (i.e. wavenumbers) that may contribute to the discrimination of the two classes of individuals, the fingerprint region (621 wavenumbers/variables) and the high region (175 wavenumbers/variables) were explored. The analysis was performed on the second derivative spectra of the two classes of samples. For the classification task we tested three different models, PCA-LDA, PLS-DA and RF, and we evaluated their performance by considering either the MS patients as a single group or split in two subgroups corresponding to the severity and duration of the disease as indicated in the previous section.

**Figure 3:**
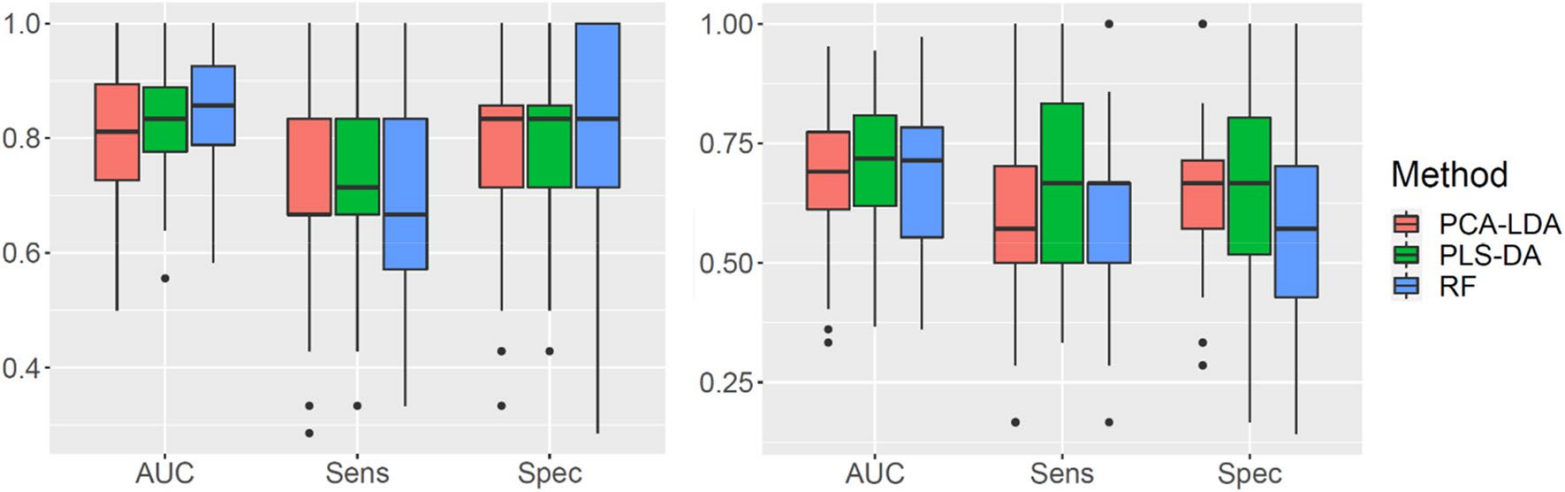
Boxplot of the area under the curve (AUC), sensitivity (Sens) and specificity (Spec) for the repeated *k*-fold cross-validation data, referred to the second derivative spectra in the two spectral regions: 1800–900 cm^−1^ (left panel) and 3050–2800 cm^−1^ (right panel). Box stretches from lower hinge (first quartiles Q1) to upper hinge (third quartiles Q3). Median is shown as a line across each box. The outliers, defined as values lower that Q1 − 1.5*IQR or upper than Q3 + 1.5*IQR, where IQR is the interquartile range, are shown as black dots. Vertical solid lines (whiskers) show the lower and upper values, excluding outliers.

For the above mentioned models, the resampling procedures described in section "Multivariate and univariate statistical analysis" were performed. The results for the three models on the 10-time repeated fivefold cross validation data, including the AUC values in the two spectral regions, are reported in Fig. 3.

Moreover, to reach a variance percentage value > 95% in the PCA-LDA model, 24 and 8 PCs are needed respectively in the analysis of the fingerprint and high region. The number of latent variables in PLS-DA and the number of variables considered at each split time in RF are equal to 3 and 35 in the fingerprint and to 1 and 165 in the high region, respectively.

A complete assessment on the performance of the final models on the test set for the three multivariate methods in terms of sensitivity and specificity is given in Table 2. The sensitivity corresponds to the probability that the test result is positive when the disease is present, whereas the specificity indicates the probability that the test result is negative when disease is not present.

**Table 2:** Sensitivity and Specificity for the chemometric models for either the MS group as a whole or separated in subgroups with different EDSS values or disease onset in the two spectral regions, 1800–900 cm^−1^ and 3050–2800 cm^−1^.

	Model	1800–900 cm^−1^		3050–2800 cm^−1^	
		Sensitivity (%)	Specificity (%)	Sensitivity (%)	Specificity (%)
	PLS-DA	67	67	67	83
	PCA-LDA	67	67	78	75
	RF	78	83	78	58
EDSS					
0.5–3.0	PLS-DA	67	63	67	75
	PCA-LDA	67	63	78	75
	RF	78	75	78	63
3.5–7.0	PLS-DA	67	75	67	100
	PCA-LDA	67	75	78	75
	RF	78	100	78	50
Disease onset					
≤ 10 years	PLS-DA	67	60	67	80
	PCA-LDA	67	60	78	80
	RF	78	60	78	60
˃ 10 years	PLS-DA	67	71	67	86
	PCA-LDA	67	71	78	71
	RF	78	100	78	57

**Figure 4:**
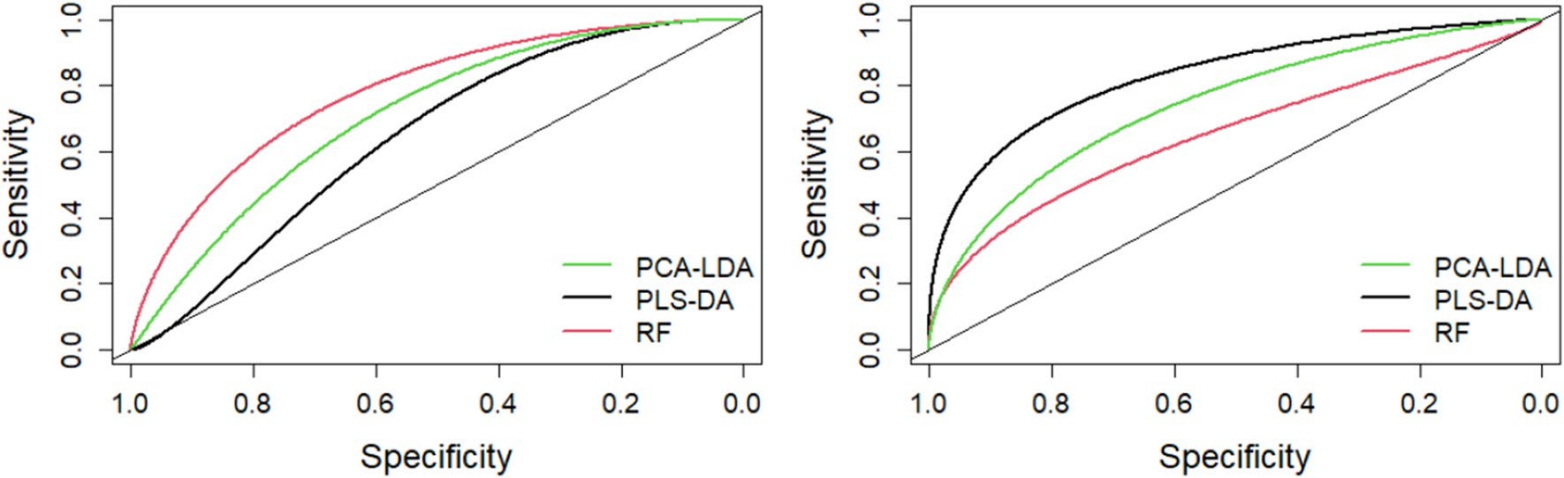
Performance measurement of the chemometric methods as described by a smoothed ROC curve considering the 1800–900 cm^−1^ (left panel) and 3050–2800 cm^−1^ (right panel) spectral regions.

Overall, the best classification in terms of sensitivity (78%) and specificity (83%) for the fingerprint region is obtained with the RF model (Table 2), whereas for the 3050–2800 cm^−1^ region the best performance is obtained with PLS-DA model. This model gives the same accuracy (0.7619) of PCA-LDA but has higher AUC value (see Fig. 4). The specificity of RF and PLS-DA models has the same value in both spectral regions, however the sensitivity is higher for the fingerprint region.

Moreover, p-values obtained from a permutation test (see, for example^53,54^), based on 10^3^ repetitions, to assess the significance against the null hypothesis of no prediction accuracy, confirm these findings both in the fingerprint region (PLS-DA: p-value = 0.043; RF: p-value = 0.001; PCA-LDA: p-value = 0.044) and high region (PLS-DA: p-value = 0.011; RF: p-value = 0.066; PCA-LDA: p-value = 0.004).

The accuracy of the statistical models and their ability to discriminate between the two groups of subjects was finally evaluated on the test set by plotting the smoothed ROC curve, which correlates the sensitivity to the specificity of the diagnostic/decisional test. Figure 4 reports the results obtained in the two regions. The higher AUC value calculated from the plot is 0.78 for the RF model in the fingerprint region (left panel), and further increases to 0.83 for the PLS-DA model in the high region (right panel). The results testify a good accuracy of these method in terms of predictive power. Considering the subtle spectral differences seen between the HC and MS spectra, the level of sensitivity and specificity obtained by our classification models is particularly remarkable for a number of reasons. In fact, the changes in the biochemical composition of the plasma are relatively minor compared to changes in CSF and do not necessary reflect all possible alterations occurring in the CNS during the progression of the MS disease, which affect different functional domains. However, despite the challenge in obtaining a more favorable sensitivity, in some recent studies a number of molecular biomarkers for MS disease identified in both plasma and CSF were proposed^8,55^. For comparison, AUC values of 0.86 are reported by an FTIR study on the CSF aiming to separate patients with clinically isolated syndrome from RRMS patients^22^, whereas slightly lower AUC values (0.82–0.83) where obtained using NMR data^56^. Serum-based differentiation among MS, amyotrophic lateral sclerosis and healthy controls was proposed by using RF method applied to the fingerprint region of the FTIR spectra. In this pilot study, the 32 MS patients included in the analysis have EDSS values in the 4.0–7.5 range (moderate/severe range). The results show a good performance of the model in discriminating healthy from pathological serum (overall precision is 83.3% for the test data set) whereas the approach is less sensitive in distinguishing the two diseases^38^.

**Figure 5:**
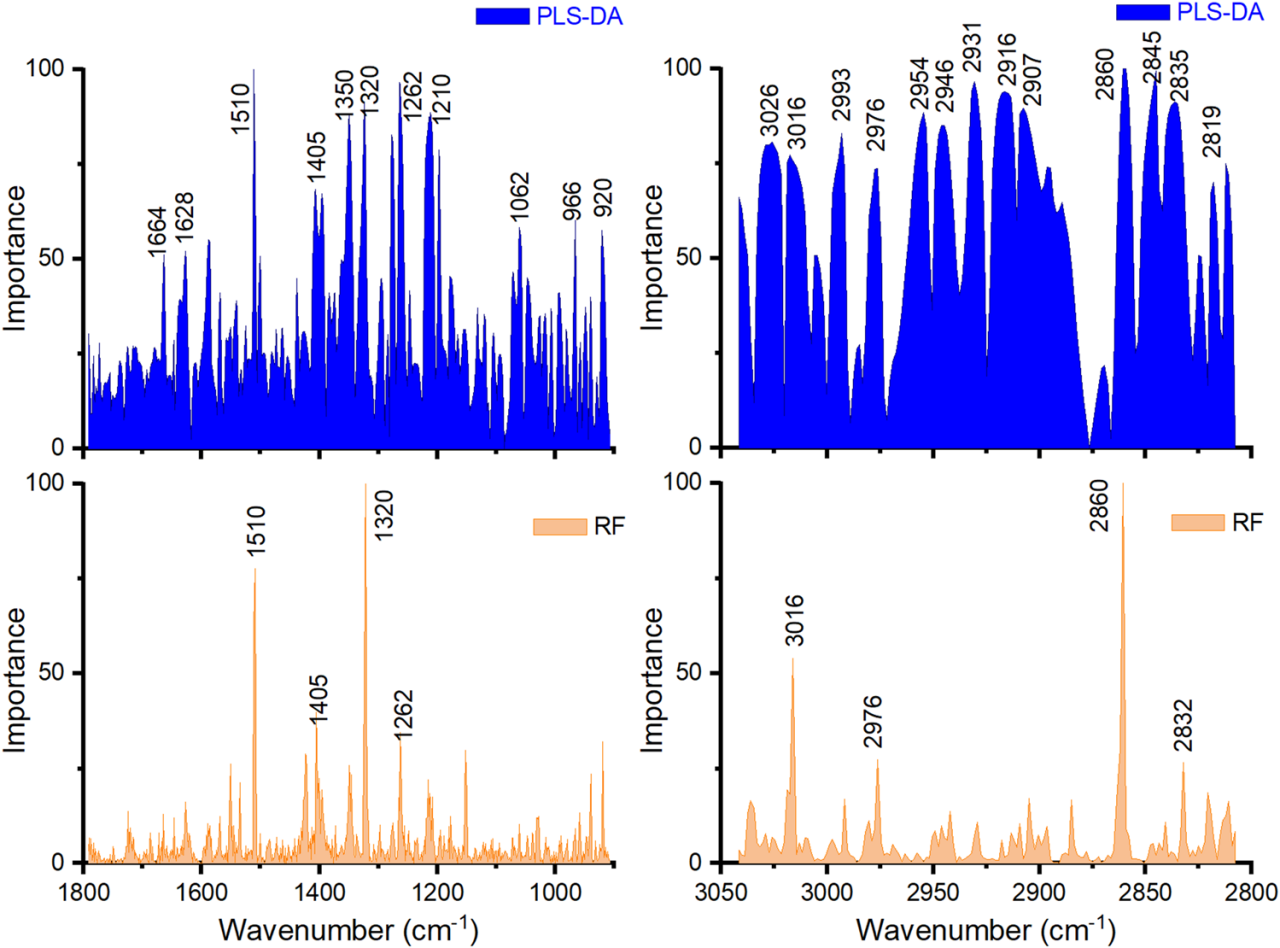
Wavenumbers importance profiles for the discrimination of HC from MS subjects by using the PLS-DA and RF methods on the second derivative spectra.

In Fig. 5 are compared the wavenumbers importance profiles obtained by using PLS-DA and RF methods in the 1800–900 cm^−1^ and 3050–2800 cm^−1^ regions, which provide the most relevant variables responsible for the classification. The plots show that, for both regions, the RF multivariate method is more selective than PLS-DA, in terms of number of variables used/needed for discrimination. However, it is interesting to note that the top wavenumbers for RF are also present in the PLS-DA plot, and with comparable importance value. These wavenumbers can be considered as marker peaks, whose changes in intensity reflect the statistically significant molecular alteration associated with the presence of the disease. Exploring in more detail the selected peaks emerging from the plots in Fig. 5, we can find interesting association with possible pathological mechanisms. The peak at 1510 cm^−1^ is assigned to amide II, indole ring (tryptophan side chain)^37,57^. Indole rings are target susceptible to damage by reactive oxygen species under oxidative stress condition occurring in inflammatory diseases. Tryptophan, in addition to being commonly contained in many protein sequences is also a free amino acid in plasma, and has a key role in serving as a checkpoint in the activation of the immune system by the enzyme indoleamine 2,3-dioxygenase^46^. Higher level of tryptophan and glutamic acid were found in the serum of MS patients compared to healthy control, and these changes were tentatively explained as a consequence of the chronic overactivation of the immune system in the patients^49^. Moreover, also proteins containing tyrosine residues may be the target of oxidative stress and spectral features in the FTIR signals associated to their level was found lower in the serum of MS patients compared to HC individuals^39^.

The 1405 cm^−1^ peak is assigned to CH_3_ asymmetric deformation of urea/triglycerides^37^, which are usually abundant in healthy plasma; the triglycerids levels in MS patients was reported to be higher than in control subjects^42^. Other assignements are^37^: 1320–1350 cm^−1^ twisting/wagging vibrational modes of CH_2_ and CH_3_ in collagen and nucleic acids; 1262, ~ 1210 cm^−1^ ${PO}_{2}^{-}$ due to asymmetric stretching vibration (nucleic acids, phospholipids); 966 and 920 cm^−1^ due to C–O, C–C stretching of sugar in nucleic acids. Important peaks in the amide I band of proteins are selected in the PLS-DA plot, consisting in the 1664 and 1628 cm^−1^ peaks, and corresponding to solvated disordered structures and β-sheet secondary structures, respectively.

For the high region (Fig. 5, right panel), the discriminant wavenumbers in the RF plot also found in the PLS-DA are related to the C–H and N–H stretching vibration of lipids and proteins. In particular, the peak at 3016 cm^−1^ is assigned to C=CH stretching of unsaturated lipids and CH_2_ aromatic stretch of proteins side chains. The wavenumber at 2976 cm^−1^ can be associated to N–H and C–H stretching, 2860 cm^−1^ to the C–H asymmetric stretching vibration, and 2832 cm^−1^ to N–H symmetric stretching ($N{H}_{3}^{+}$) also present in free amino acids.

Overall, all these observations show that the most important wavenumbers for differentiation can be assigned to specific functional groups of molecular species, indicating significant spectral differences connected to subtle altered levels of biomolecules in the plasma.

## Conclusion

ATR-FTIR spectroscopy of easily collected body fluids represents a powerful biomolecular profiling method that can be thoroughly explored for clinical diagnostic purpose. Many efforts are ultimately directed in this direction for the diagnosis and monitoring of diseases, mainly of neurodegenerative type, which are highly disabling and difficult to identify.

The results presented in this study show that there are subtle and yet statistically significant spectral differences in the plasma of patients affected by multiple sclerosis compared to a control healthy group. In particular, we found an increased lipid/protein ratio in the MS cohort, indicating a dysregulation (either up- or down-regulation) of the protein-lipid metabolism.

Three different multivariate methods were applied on the fingerprint region and on the high region of the ATR-FTIR spectra to discriminate between pathological and healthy individuals. The results show that the RF model provides the best performance in the fingerprint region, with a sensitivity of 78% and specificity of 83%, whereas for the high region the best performance is given by the PLS-DA model. The latter model maintains the same specificity value as the RF ones, but the sensitivity decreases to 67%, indicating that the fingerprint region is more significant for an effective classification.

The discrimination ability of the method here proposed, based on ATR-FTIR spectra of plasma coupled with advanced chemometric techniques, is highly encouraging and can open new routes for additional ways for helping MS diagnosis, which requires long and costly demanding processes.

## Supplementary Information

Supplementary Information.

## Data Availability

The raw data generated and analyzed during the current study are available from the corresponding author on reasonable request.
