# Supplementary material for: ATR-FTIR spectroscopy of plasma supported by multivariate analysis discriminates multiple sclerosis disease
Source: Sci Rep. 2023 Feb 13;13:2565. doi: 10.1038/s41598-023-29617-6 (PMC9924868; doi:10.1038/s41598-023-29617-6)
Supplement: Supplementary file 1 — Supplementary Information. [file 41598_2023_29617_MOESM1_ESM.pdf]

Supporting Information

ATR-FTIR spectroscopy of plasma supported by multivariate analysis discriminates multiple sclerosis disease

Maria Caterina Crocco[1,2], Maria Fernanda Heredia Moyano[1], Fernanda Annesi[3], Rosalinda Bruno[4], Domenico Pirritano[5], Francesco Del Giudice[5], Alfredo Petrone[5], Francesca Condino[6], Rita Guzzi[1,3,]*

[1]Department of Physics, Molecular Biophysics Laboratory, University of Calabria, 87036 Rende, Italy
[2]STAR Research Infrastructure, University of Calabria, Via Tito Flavio, 87036 Rende (CS), Italy
[3]CNR-NANOTEC, Licryl-UOS Cosenza and CEMIF.Cal, Department of Physics, University of Calabria, 87036 Rende, Italy
[4]Department of Pharmacy, Health and Nutritional Sciences, University of Calabria, 87036 Rende (CS), Italy;
[5]Neurological and Stroke Unit, Multiple Sclerosis Clinic, Annunziata Hospital, 87100 Cosenza, Italy
[6]Department of Economics, Statistics and Finance "Giovanni Anania", University of Calabria, Arcavacata di Rende, CS, Italy

Corresponding author: Rita Guzzi (rita.guzzi@fis.unical.it)

Table S1: Band assignments of the main peak absorption of plasma in the 3050 – 900 cm^{-1} IR region [1–4]	
Wavenumber (cm^{-1})	Functional groups assignment
3010	Olefinic C= CH stretching vibration (unsaturated lipids)
2957	CH_3 asymmetric stretching (lipids and proteins)
2923	CH_2 asymmetric stretching (lipids and proteins)
2872	CH_3 symmetric stretching (proteins and lipids)
2852	CH_2 symmetric stretching (lipids and proteins)
1739	C=O stretching (ester functional groups in lipids, fatty acids, cholesterol, triglycerides)
1650	Amide I (protein C=O stretching)
1546	Amide II (protein NH bending, CN stretching)
1480-1430	CH_3, CH_2 bending (mainly lipids)
1399	COO^- symmetric stretching (fatty acids, amino acids)
1337	CH_3 symmetric bending (lipids)
1315	CH_2 wagging (lipids)
1240	PO_2^- asymmetric stretching (nucleic acids, phospholipids)
1173	CO – O – C asymmetric stretching (ester bonds in cholesteryl esters)
1170-1120	C– O and C– O –C stretching (polysaccharides, glycogen)
971	$C–N^{\pm}–C$ stretching (nucleic acids)
924	Ribose ring vibrations (RNA)

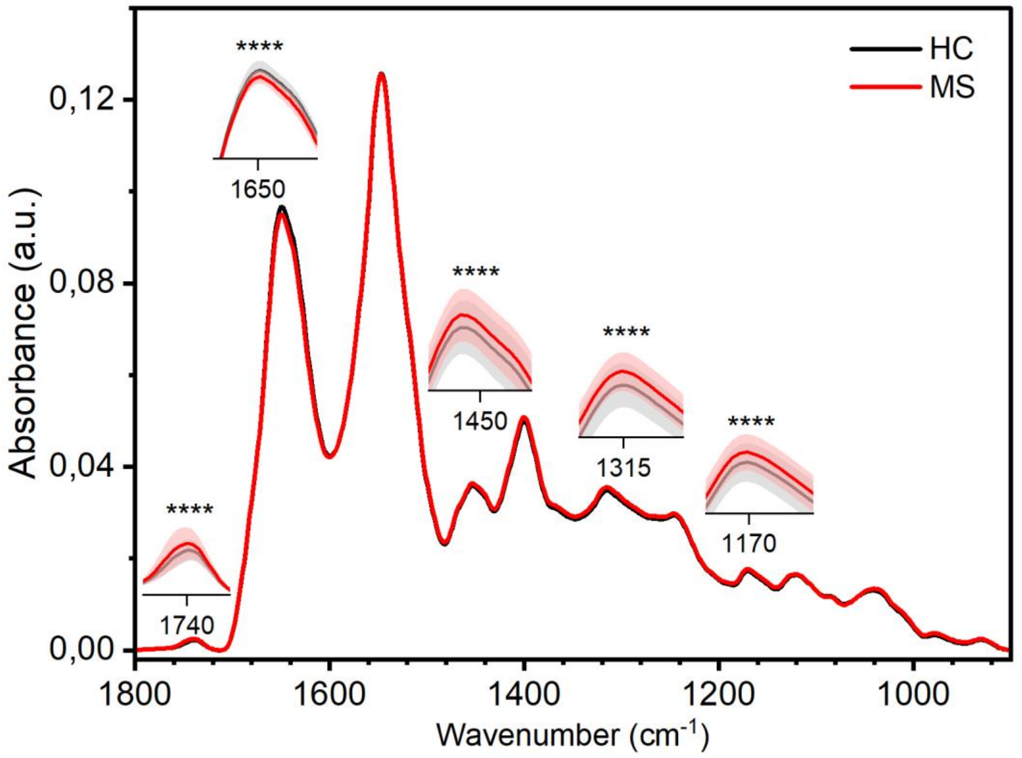

Figure S1 – Class means with standard deviation for HC and MS subjects in the 1800-900 cm^{-1} fingerprint region. Preprocessed steps on the spectra are: cut, rubber band baseline, vector normalization (see main text for details). Selected spectral regions are enlarged and the statistical significance is shown according to their p value (****$p < 0.0001$).

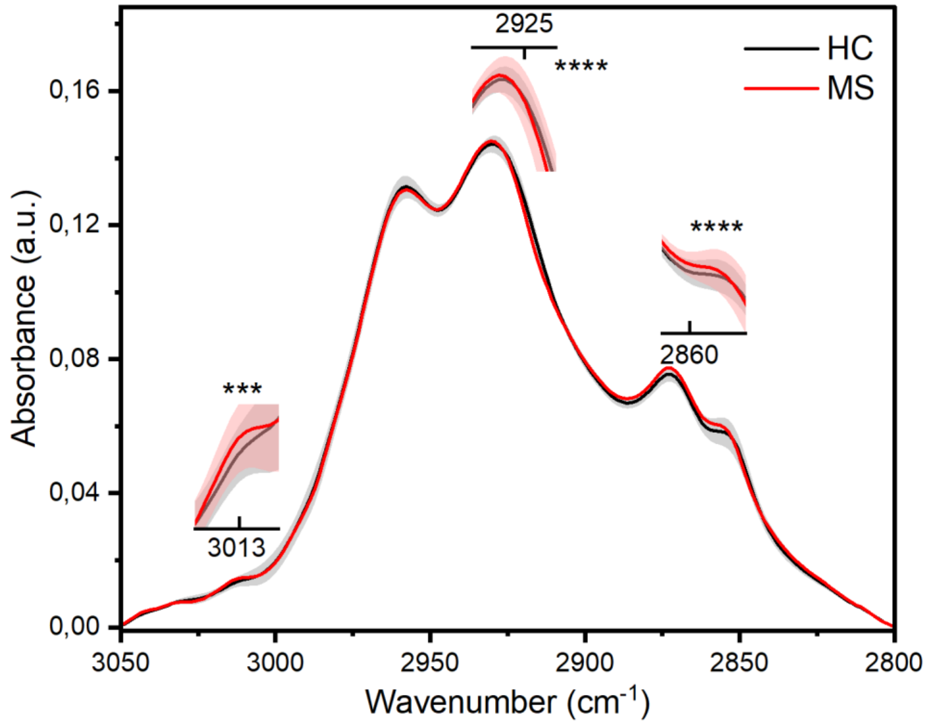

Figure S2 – Class means with standard deviation for 40 HC and 45 MS subjects in the 3050-2800 cm^{-1} region. Preprocessed steps on the spectra are: cut, rubber band baseline, vector normalization. Selected spectral regions are enlarged and the statistical significance is shown according to their p value (***p < 0.001; ****p<0.0001).

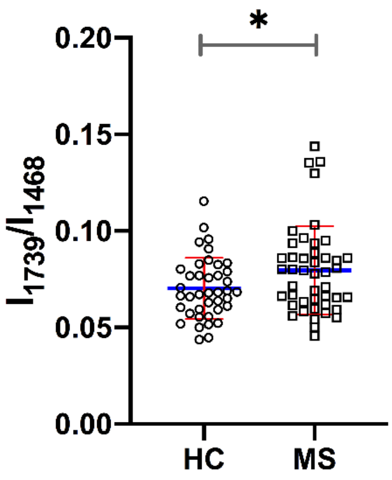

Figure S3 – Ratio of the intensities of the carbonyl C=O peak at 1739 cm^{-1} and of the CH_2 scissoring motion at 1446 cm^{-1} in the spectra obtained for HC and MS. The blue lines represent the mean values and the red lines the standard deviation. The degree of significance of the comparison between HC and MS is indicated as *p<0.05

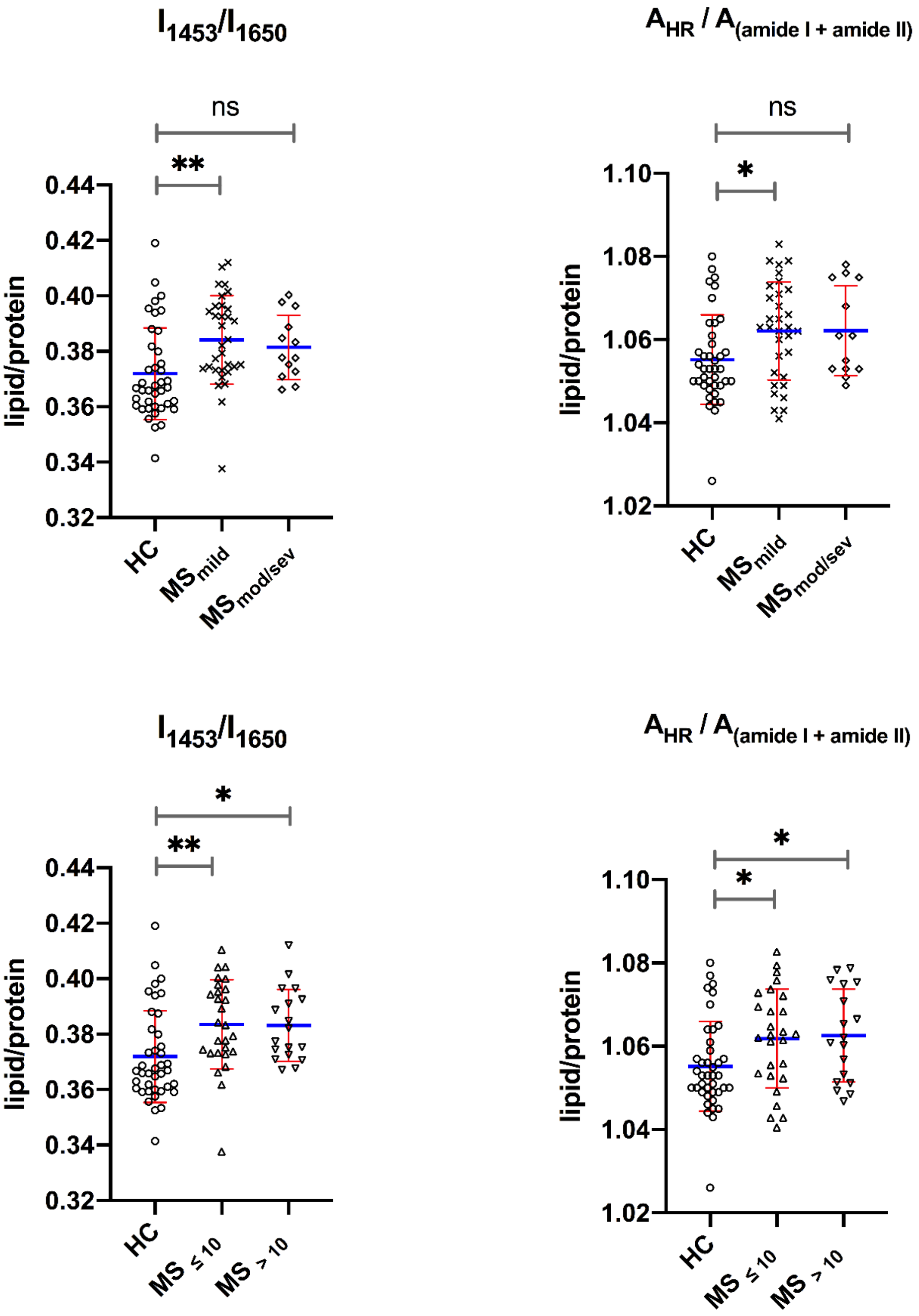
I1453/I1650
ns
**
lipid/protein
0.44
0.42
0.40
0.38
0.36
0.34
0.32
HC
MS mild
MS mod/sev
AHR / A(amide I + amide II)
ns
*
lipid/protein
1.10
1.08
1.06
1.04
1.02
HC
MS mild
MS mod/sev
I1453/I1650
*
**
lipid/protein
0.44
0.42
0.40
0.38
0.36
0.34
0.32
HC
MS ≤ 10
MS > 10
AHR / A(amide I + amide II)
*
*
lipid/protein
1.10
1.08
1.06
1.04
1.02
HC
MS ≤ 10
MS > 10

Figure S4 – Lipid/protein ratio for HC and MS subgroups separated according to EDSS score and disease duration. The data show the ratio of the intensity of the peaks at 1453 cm^{-1} to the amide I at 1650 cm^{-1} (left panel) and the ratio between the areas under the high region (3050-2800 cm^{-1}) and the sum under the amide I and amide II bands (right panel). The blue lines represent the mean values and the red lines the standard deviation. The degree of significance of the comparison between HC and MS is indicated as *$p<0.05$; **$p < 0.01$; "ns" stands for "not significant".

References

1. Baker, M. J. *et al.* Developing and understanding biofluid vibrational spectroscopy: a critical review. *Chem. Soc. Rev.* **45**, 1803–1818 (2016).
2. Rehman, I. ur, Movasaghi, Z. & Rehman, S. FTIR and Raman Characteristic Peak Frequencies in Biological Studies. in *Vibrational Spectroscopy for Tissue Analysis* (CRC Press, 2012).
3. Stuart. Biological Applications. in *Infrared Spectroscopy: Fundamentals and Applications* 137–165 (John Wiley & Sons, Ltd, 2004). doi:10.1002/0470011149.ch7.
4. Tamm, L. K. & Tatulian, S. A. Infrared spectroscopy of proteins and peptides in lipid bilayers. *Q Rev Biophys* **30**, 365–429 (1997).
